# “Peritoneal failure”: A new concept to explain negative results of randomized trials evaluating intraperitoneal therapies

**DOI:** 10.1515/pp-2020-0117

**Published:** 2020-05-19

**Authors:** Marc Pocard, Marc A. Reymond

**Affiliations:** Université de Paris, INSERM UMR 1275 CAP Paris-Tech, F-75010 Paris, France; National Center for Pleura and Peritoneum (NCPP) Tübingen University, Tübingen 72076, Germany; Service de chirurgie digestive et cancérologique Hôpital Lariboisière 2 rue Ambroise Paré, F-75010 Paris, France

Prof. Jimmy So recently presented the results of the Extensive Peritoneal Lavage after curative gastrectomy for gastric cancer (EXPEL) study at the Gastrointestinal Cancers Symposium 2020 (ASCO GI) in San Francisco [1]. The EXPEL trial is a prospective randomized, high-quality surgical study evaluating the potential benefit of peritoneal lavage after surgical resection of the stomach. The trial involved 800 patients from 22 hospitals from Korea, China, Japan, Malaysia, and Singapore. Patients with cT3, T4 stomach cancer undergoing curative resection were randomized to surgery alone (control group, n=402 patients) or surgery followed by lavage of the peritoneal cavity with 10 L of saline solution (test group, n=398 patients). There was no difference in the 3-year cumulative incidence of recurrence between the two groups. The rate of adverse events was higher in the test group (RR=1.58, P=0.019).

The EXPEL study is not the first study failing to show a benefit of intraperitoneal therapies. Since 2018, the community of peritoneal surgeons is collecting negative results with hyperthermic intraperitoneal chemotherapy (HIPEC) in colorectal cancer, both in the prophylactic and in the therapeutic setting [2, 3]. Positive results talk louder than negative studies. They are more appealing to physicians and get broader coverage. But does that mean that peritoneal surgeons should be discouraged from pursuing a fruitless path? Or might these negative studies give them valuable insights into where to look next?

Many comments have been made regarding the failure of the French PRODIGE 7 randomized controlled trial examining an additional benefit of HIPEC over cytoreductive surgery alone in patients with peritoneal metastasis of colon cancer [4, 5, 6]. The PRODIGE 7 trial showed a remarkable overall survival of around 41 months in both groups, and the control group (surgery alone) performed much better than expected. Thus, the additional effect of HIPEC, if any, was too small to be detected with the sample size available. Moreover, the HIPEC effect, if any, was erased by the increased postoperative morbidity in the test group.

The reasons for these repeated, unexpected failures of intraperitoneal therapies in clinical trials might differ between trials, but some lessons can be learned for all of them.

The first lesson is methodological and might appear self-evident. Medical research does not start with Phase-3 trials. Peritoneal surgeons should first go back to the laboratory to explore new approaches such as advanced drug delivery systems, nanoparticles, carrier solutions, and others. Only a few of these approaches will go successfully through the preclinical development steps and will reach clinical testing in human patients. These new approaches should then be validated step by step in well-designed Phase-I and (controlled) Phase-II trials. Out of the strategies tested in early-phase clinical trials, only the most promising will make it to Phase-3 trials, with increased chances of success.

The second reason is biological, and we would like to introduce, for the first time, the concept of “peritoneal failure”. Peritoneal failure is defined as the loss of function(s) of the organ peritoneum. When treating patients, peritoneal surgeons should protect the protector, the peritoneum, which is the first and highly effective line of defense against aggression. Intraperitoneal therapies should decrease tumor cell viability or combat effectively bacterial pathogens. They should indeed also preserve the function of the peritoneal membrane and the associated immunological structures. Like the abdominal skin, the peritoneum derives embryologically from the lateral plate of the mesoderm [7]. Like the skin, the peritoneum has extraordinary developed immunological properties [8]. The most effective way to impair the immune response of the peritoneum is to damage and destroy it.

The hypothesis of the EXPEL trial [1] was that extensive intraoperative peritoneal lavage (EIPL) would decrease tumor cell implantation in the absence of macroscopic peritoneal metastasis. Earlier publications documented that cancer cell spilling is an ordinary happening during cancer surgery and that these cells can grow [9]. Moreover, most cells shed into the peritoneal cavity undergo spontaneous death, the so-called anoikis [10]. Since abundant peritoneal lavage is detaching tumor cells from the extracellular matrix, and isolating them from other cells such as cancer-associated fibroblasts, it should promote anoikis and intraperitoneal cell death. Thus, the rationale of EXPEL trial was robust. However, the clinical trial failed to meet the expectations. Why?

We can find some explanations in the past. Thirty years ago, Moshe Shein proposed extensive peritoneal lavage to improve the outcome of severe bacterial peritonitis. Like the EXPEL study in cancer, a randomized trial failed to show any clinical benefit of extensive peritoneal lavage for treating severe bacterial peritonitis [11]. Is there a common explanation for both benign and malignant situations? Probably yes. Extensive lavage clears the peritoneal cavity not only from bacteria (or tumor cells) but also from lymphocytes and macrophages. In the peritonitis and in the cancer situation, the impaired adaptive immune response of the organ peritoneum erased the positive effects of bacterial, respectively, tumor cell clearance. Another example is the intraoperative peritoneal irrigation with saline solution after cesarean sections. Many obstetricians performed peritoneal irrigation routinely until results from clinical trials delivered conflicting results on its benefit. In one trial, irrigation at cesarean delivery increased intraoperative nausea without decreasing postoperative infectious morbidity [12].

Prior science has pointed out that aggression to the peritoneum can favor disease progression. For example, the exposition of the peritoneum to cold, dry CO_2_ promotes tumor cell implantation [13, 14]. A characteristic feature of the negative clinical trials on intraperitoneal therapies is that they all used aggressive protocols. The PRODIGE 7 trial used HIPEC with the highest tolerated Oxaliplatin concentration (460 mg/m^2^) and the highest tolerable temperature (43 °C). The EXPEL trial used “extensive” peritoneal lavage with 10-L saline solution. Did all these aggressive procedures cause a failure of the organ peritoneum?

The Dutch trial on ovarian cancer is the only randomized study showing an additional benefit of HIPEC over cytoreductive surgery alone [15]. Interestingly, this trial used only limited hyperthermia at 40° with lower drug exposure but with prolonged HIPEC procedure. This study did not report an increase in postoperative morbidity. Such “organ-preserving” approaches might be the best option because they reach an optimal balance between local efficacy and toxicity. For this reason, the International Society for the Study of Pleura and Peritoneum (ISSPP) has awarded the first author of this study, Dr Willemien J. van Driel with its Peritoneum Prize 2019 [16].

We should now learn the lessons of the negative trials on intraperitoneal therapies in the past or we will fail again in the next trial. “Peritoneal failure” appears to be the crucial concept beyond these negative results. We need to develop therapeutic protocols taking into account the peritoneal cavity as a unique therapeutic and immunological space. We should protect as far as possible the multiple functions of the organ peritoneum. We need to gather more information from fundamental research. We should use validated methodology to translate this basic information, step by step, into clinical reality. The observance of these principles might considerably increase the chances of successful clinical innovation in peritoneal diseases.

## Notes

**Marc Pocard**, Université de Paris, INSERM UMR 1275 CAP Paris-Tech, F-75010 Paris, France; Service de chirurgie digestive et cancérologique Hôpital Lariboisière 2 rue Ambroise Paré, F-75010 Paris, France, E-mail: marc.pocard@gmail.com **Marc A. Reymond**, National Center for Pleura and Peritoneum (NCPP) Tübingen University, Tübingen 72076, Germany, E-mail: marc.reymond@yahoo.de
